# Perioperative nursing care for a patient undergoing image-guided planned autologous islet transplantation after total pancreatectomy: A case report

**DOI:** 10.1097/MD.0000000000049965

**Published:** 2026-07-24

**Authors:** Jianwen Yang, Xuefeng Qi, Qin Xu, Weiming Qian

**Affiliations:** a Nursing Department, The Second Affiliated Hospital of Zhejiang University School of Medicine, Hangzhou, Zhejiang, China.

**Keywords:** autologous islet cell transplantation, image-guided, planned surgery, total pancreatectomy

## Abstract

**Rationale:**

Total pancreatectomy (TP) often leads to insulin-deficient diabetes, including unstable “brittle diabetes.” Autologous islet transplantation during or after TP can prevent or treat diabetes by reinfusing functional islets into the portal vein. This case report details the perioperative nursing strategies for a patient undergoing image-guided planned autologous islet transplantation following TP.

**Patient concerns:**

A 64-year-old male with pancreatic cancer, type 2 diabetes, and hypertension underwent TP and initial autologous islet transplantation but required a second transplantation due to poor graft function. Preoperatively, he exhibited fluctuating blood glucose (8.9–14.5 mmol/L) and anxiety about surgical outcomes.

**Diagnoses:**

The patient was diagnosed with recurrent pancreatic cancer post-TP and chemotherapy, compounded by insulin-dependent diabetes and suboptimal islet function necessitating secondary transplantation.

**Interventions:**

A multidisciplinary approach included meticulous preoperative islet quality control, intraoperative portal vein catheterization under ultrasound guidance, and dynamic monitoring of portal pressure. Postoperative care featured anticoagulation (heparin/low-molecular-weight heparin), glycemic management via insulin micropump, and thromboprophylaxis using intermittent pneumatic compression. Psychological support and preoperative education alleviated patient anxiety.

**Outcomes:**

Posttransplantation, blood glucose stabilized (3.9–7.7 mmol/L), enabling discontinuation of exogenous insulin. The patient was discharged on day 18 without complications such as thrombosis or hemorrhage.

**Lessons:**

Multidisciplinary collaboration, tailored nursing interventions, and rigorous complication prevention ensured successful outcomes. This case highlights the critical role of structured perioperative care in achieving insulin independence after complex islet transplantation.

## 1. Introduction

After total pancreatectomy (TP), patients may develop insulin-deficient diabetes, and some patients exhibit “brittle diabetes,” characterized by large fluctuations and extreme instability in blood glucose levels.^[1]^ By preparing the resected pancreas to collect autologous islets with normal endocrine function and reinfusing them into the body, diabetes related to pancreatectomy can be prevented and treated. Autologous islet cell transplantation is usually performed concurrently with the pancreatectomy.^[2]^ The portal vein is the preferred implantation site for islet cell transplantation,^[3]^ leveraging its rich blood supply to promote cell survival and functional expression. Islet cell transplantation is a minimally invasive and relatively safe treatment, and its efficacy in diabetes has been widely demonstrated.^[4]^ In May 2024, our hospital conducted image-guided planned autologous islet cell transplantation for a patient after total pancreatectomy, enabling the patient to become insulin-independent. After careful treatment and nursing, the patient recovered and was discharged from the hospital after 18 days of hospitalization. The patient has ceased exogenous insulin therapy. The surgical nursing experience is reported below.

## 2. Patient concerns and diagnosis

This study was approved by the Ethics Committee of The Second Affiliated Hospital of Zhejiang University School of Medicine (Approval No. 2024-0897). The patient is a 64-year-old male with a history of type 2 diabetes and hypertension. In November 2023, he presented with epigastric pain and visited a local hospital, where he was diagnosed with pancreatic cancer. He subsequently underwent 3 cycles of albumin paclitaxel combined with gemcitabine regimen (AG regimen) at the local hospital. Then, in December 2023, the patient underwent a total pancreatectomy, autologous islet cell transplantation via the portal vein, splenectomy, portal vein reconstruction, high biliary tract reconstruction, peripancreatic neurectomy, and cholecystectomy at our hospital. The postoperative pathological report indicated a moderately to poorly differentiated ductal adenocarcinoma. Following the surgery, the patient continued to receive 5 additional cycles of the AG regimen, with the most recent cycle occurring on April 30, 2024. Currently, the patient’s general health status is stable, and he is preparing for further treatment for “pancreatic cancer.”

The patient has a history of diabetes for over ten years. Prior to the total pancreatectomy, he used insulin aspart (4 units in the morning, afternoon, and evening) and long-acting Lantus insulin (10 units before bedtime) to control his blood glucose levels. After the surgery, the patient switched to insulin aspart injections, 3 times a day, with 8 units each time, resulting in good blood glucose control. Due to the severity of the patient’s diabetes and poor islet function, it was planned to perform ex vivo expansion and reinfusion of islet cells after the patient recovered from the surgery to better control his diabetes. The patient’s random blood glucose levels fluctuated between 8.9 and 14.5 mmol/L on the day before the surgery.

On May 20, 2024, the patient underwent a second image-guided surgery, namely portal vein islet cell transplantation and hepatic portal venography. After routine disinfection and draping in the hybrid operating room, successful ultrasound-guided puncture of a branch of the portal vein segment V was performed. A 4F MPA1 catheter was used for portal vein trunk angiography, which showed patent and smooth visualization of the portal vein trunk and branches. The islet cell suspension was then reinfused through the catheter. After the reinfusion, ONYX glue, 710–1000 μm gelatin sponge particles, and coils were used to occlude the portal vein puncture tract. The surgical procedure was uneventful. Postoperative vital signs were stable, and the puncture site was properly dressed and bandaged upon completion of the surgery. The patient was safely transferred back to the ward.

Patient outcome after surgery: The patient’s random blood glucose levels fluctuated between 3.9 and 7.7 mmol/L on the day before discharge.

## 3. Interventions

This research was reviewed and approved by the Second Affiliated Hospital of Zhejiang University School of Medicine (IRB-2024-0897). Our hospital possesses a professional islet cell laboratory within the operating room. To ensure the accurate infusion of islet cells, we have developed the following detailed protocols:

### 3.1. Preoperative preparation phase

Prior to autologous islet transplantation after ex vivo expansion, patients undergo a comprehensive evaluation. This includes testing for blood glucose, insulin, C-peptide levels, glycated hemoglobin, and diabetes-related antibodies, as well as assessments of nutritional status, ophthalmology, neurology, cardiovascular and cerebrovascular health, and liver and kidney function. Additionally, doctors evaluate the patient’s neurological, cardiopulmonary, digestive, hematological, endocrine systems, and tumor indicators. Once the evaluation is completed, patients are required to sign an informed consent form.

Next, the preparation of islet cells begins. The cells undergo tests for viability and quantity, followed by cleaning and loading into a 100–300 mL infusion bag. Regular heparin is added at 50–70 units/kg of body weight. Once the cells arrive at the hospital (autologous islet cells do not require blood type or immune matching), they undergo quality inspection, including cell counting and viability testing. Samples are retained for microbial testing to ensure that the expanded islet cells are sterile and uncontaminated before use. Common disposable sterile items include an 11-gauge scalpel blade, 1 mL, 10 mL, and 20 mL syringes, a sterile infusion set without a filter, a 3-way stopcock, and extension tubing.

### 3.2. Transplant surgery phase

On the day of surgery, patients are required to take 400 mg of pentoxifylline orally and receive an intravenous infusion of 2 grams of cefmetazole sodium for anti-infective treatment before the operation. Under general anesthesia, the surgeon performs portal vein catheterization via a percutaneous transhepatic or laparoscopic approach. One hour prior to the infusion of islet cells, patients receive a 50 mg intravenous injection of etanercept, along with 25 mg of promethazine as an anti-allergic medication. To prevent thrombosis, systemic anticoagulant therapy is also administered, and portal vein pressure is monitored during the infusion to ensure it remains below 22 mm Hg.

During the infusion, the infusion bag containing the islet cells is shaken repeatedly every 3 minutes to prevent precipitation of the islet cells and related tissues, which could affect their reinfusion. After the completion of the infusion, the puncture path is sealed using Onyx glue, liver coils, and anhydrous ethanol. Following this, the patient is transferred to the intensive care unit for close observation.

### 3.3. Posttransplantation management phase

Within 48 hours after surgery, patients receive heparin sodium anticoagulation therapy, administered as a continuous infusion of 12,500 units plus 50 mL of normal saline, maintaining an activated partial thromboplastin time (APTT) increase of 10–15 seconds. Within a week, this is switched to a subcutaneous injection of 30 mg (3000 units) of low-molecular-weight heparin until Doppler ultrasonography of the liver shows normal portal vein blood flow. From the second day onward, patients are also prescribed a small dose of 100 mg of aspirin enteric-coated tablets.

On the day of transplantation, insulin is continuously infused via a micro-pump, with the goal of maintaining fasting blood glucose levels below 6.7 mmol/L. Within 5 days after surgery, the control of blood glucose levels is closely related to the withdrawal of insulin. For 1 month, a low dose of insulin is maintained to protect the newly transplanted islets from stress. Within 3 months, blood glucose levels are strictly controlled to be below 7.8 mmol/L fasting, preferably between 4.5–6.4 mmol/L, and below 10 mmol/L 2 hours after meals. Additionally, patients are prescribed 400 mg of pentoxifylline for 7 days and receive subcutaneous injections of 5 mg of etanercept on the third, seventh, and 10 days after surgery. Other medications such as anti-infectives, liver protectants, stomach protectants, nutritional support, and fluid replenishment are also necessary.

Portal vein blood flow is monitored at 12 hours, 1 day, 3 days, 1 week, 2 weeks, 1 month, 3 months, 6 months, and 1 year after surgery to monitor blood flow velocity and prevent portal hypertension. Regular follow-ups are scheduled at 1 month, 3 months, 6 months, and 1 year after surgery to check glycated hemoglobin, blood glucose levels, islet function, and nutritional status.

## 4. Complication nursing

### 4.1. Effectively alleviating anxiety about a second planned surgery

One day before the surgery, the operating room nurse and the primary nurse jointly reviewed the electronic medical record to understand the patient’s underlying diseases, medication history, coagulation time, blood glucose control, and other relevant information. They employed the CICARE communication model^[5]^ to identify the primary concerns of the patient and their family, which primarily centered on doubts about the treatment outcome. The nurses patiently explained the latest advancements in islet cell transplantation and how the surgery precisely delivers islet cells into the patient’s hepatic portal vein under ultrasound and image-guided guidance.

Addressing the patient’s worry about not being able to tolerate the surgical procedure, the nurses patiently instructed them on relaxation techniques, such as deep breathing exercises, and explained that this surgical approach is particularly suitable for patients with poor clinical conditions, high surgical risks, and those who cannot tolerate traditional surgical treatments, offering advantages such as minimal trauma and rapid recovery. Through active and effective communication, the patient and their family agreed to undergo the surgery and gained a clearer understanding of the procedure, which helped alleviate their anxiety to some extent. This reduction in anxiety was reflected in the Hamilton Anxiety Scale (HAMA) scores,^[6]^ which decreased from 36 points (severe anxiety) to 8 points (mild anxiety).

### 4.2. Establishing a hemorrhage emergency plan

A multidisciplinary discussion is conducted preoperatively to determine the emergency response plan. In the event of unexplained hepatic portal vein hemorrhage, the primary surgeon should be immediately notified, and ultrasound or other imaging techniques should be utilized to confirm whether the hemorrhage is caused by catheter displacement or detachment. Once it is confirmed that the hemorrhage is due to a catheter-related issue, the emergency response team should be swiftly activated, and preparations should be made for an emergency laparotomy to repair or stop the bleeding. Before surgery, nurses need to prepare the instruments required for laparotomy, including but not limited to laparotomy instruments, liver retractors, hemostatic forceps, sutures, and other materials, and ensure comprehensive sterilization of the surgical area, extending from the level of the nipples to the pubic symphysis and laterally to the mid-axillary lines. Intraoperatively, close monitoring of the patient’s digital subtraction angiography (DSA) images and other multimodal imaging changes is essential to assess the specific location and volume of bleeding. Any manipulation should be preceded by a careful assessment of its stability and risk of hemorrhage. In this case, the patient did not experience such complications.

Before surgery, nurses conduct a comprehensive assessment of patients, providing psychological support and health education to help them prepare physically and mentally. During the procedure, they strictly adhere to aseptic principles and play a vital role in assisting the surgical team, including continuously observing catheter placement guided by intraoperative ultrasound, monitoring for complications, and tracking vital signs – particularly blood pressure and heart rate – to promptly detect signs of bleeding. Although percutaneous or laparoscopic surgery is preferred, both the circulating and scrub nurses must be prepared at all times to assist in converting to open laparotomy should intraoperative emergencies such as massive hemorrhage, perforation, or other critical complications arise, ensuring the safe and successful transplantation of islet cells. After surgery, nurses continue their critical role by closely monitoring vital signs, blood glucose levels, and potential complications such as infection, bleeding, or portal vein thrombosis. They also implement pain management, nutritional support, and medication care, while providing patients with comprehensive health education on insulin use, dietary guidance, and necessary lifestyle adjustments.

### 4.3. Intraoperative thrombosis prevention and response

Studies have shown^[7]^ that TP (presumably referring to a specific procedure or treatment not fully explained in the context, but likely related to islet transplantation) and the reinjection of the patient’s islets into the portal vein can trigger a local inflammatory response and increased portal vein pressure, threatening islet survival and potentially leading to portal vein thrombosis. Therefore, intraoperative dynamic monitoring of indices such as the average portal vein blood flow velocity, portal vein blood flow volume, hepatic artery resistance, and diastolic hepatic artery flow is crucial for assessing liver blood circulation after transplantation and for early detection of portal vein thrombosis.^[8]^

Addressing the potential risks of deep vein thrombosis (DVT) in the lower extremities and venous thromboembolism (VTE), the operating room nursing team implemented a series of preventive measures. VTE is one of the common complications of surgical procedures, with an incidence rate of up to 10% to 40% in general surgical patients when preventive measures are not taken.^[9]^ In view of this, the operating room nursing team utilized the VTE risk assessment tool (Caprini model) to evaluate the patient’s VTE risk. In this case, the patient’s postoperative VTE risk score was 7, categorizing them as high-risk^.[10]^ Consequently, physical preventive measures were adopted, and the operating room nursing team also incorporated intermittent pneumatic compression devices for the lower extremities^[11]^ into the preventive strategy. These devices help improve blood circulation in the lower extremities through periodic compression, reducing the opportunity for thrombus formation. Under standardized specialist nursing care, the patient in this case did not develop deep vein thrombosis in the lower extremities.

In terms of intraoperative preparation, it is crucial to ensure that sufficient thrombectomy catheters, filters, and other necessary instruments such as guidewires, catheter sheaths, and thrombolytic drug infusion systems are readily available. Additionally, medications for dissolving blood clots, such as urokinase or recombinant tissue plasminogen activator (rt-PA), should be prepared, and healthcare professionals need to be familiar with their usage and dosage adjustment principles. If conditions permit, angiography or other imaging techniques can be utilized to aid in the diagnosis and treatment of thrombosis, ensuring timely and effective management of potential thrombus situations.

The current standard for effectively addressing thrombosis involves intraoperative administration of unfractionated heparin combined with postoperative low-molecular-weight heparin for anticoagulation. However, during this process, the most common adverse reaction to anticoagulants is bleeding, which may include skin bleeding, gastrointestinal bleeding, urinary bleeding, and other types. The severity of bleeding can range from mild conditions such as skin bruises, nosebleeds, and gum bleeding to severe conditions like intracranial hemorrhage. To prevent these complications, several measures should be taken, including regular monitoring of the patient’s coagulation function (such as prothrombin time [PT], APTT, and international normalized ratio [INR]), observing the patient’s skin condition, urine appearance, and pupil changes every 15 minutes during the surgery, paying close attention to the patient’s coagulation function indicators, communicating promptly with the physician, and adjusting the dosage of anticoagulants dynamically as prescribed.

### 4.4. Nursing care for islet cell expansion and transplantation

During the nursing care process for islet cell expansion and transplantation, operating room nurses need to prepare in advance the precooled modified University of Wisconsin (UW) preservation solution, which is used for the preservation of the pancreas. Immediately after the pancreas is resected, it should be submerged in UW preservation solution, and an infusion tube is inserted into the pancreatic duct from the head of the pancreas to ensure proper preservation of the pancreatic tissue. Subsequently, the pancreas immersed in UW preservation solution is immediately transported to the islet isolation room within the operating room to prepare for subsequent islet cell expansion and isolation.

During the digestion process, the status and quantity of islets in the digestive fluid are continuously monitored to ensure the integrity and viability of the islet cells. After the completion of islet isolation, a preliminary viability test is performed on the islet cells to ensure they meet the criteria for transplantation. Following the washing and expansion treatment, the islet cells are suspended in a 200 to 500 milliliter solution of human serum albumin to prepare an islet cell suspension, which is then placed in a sterile bag. The patient’s islet cell expansion solution is stored appropriately, and the outer packaging is clearly labeled with the patient’s name, gender, age, medical record number, bed number, blood type, diagnosis, specimen name, specimen dose, and expiration date. This ensures that each sample has a clear label for easy management and tracking.

The operating room nurse needs to follow medical instructions and place the islet cell expansion solution at room temperature 2 hours before surgery, allowing it to naturally warm up to near room temperature. This step reduces the risk of intraoperative hypothermia and decreases the incidence of complications. After the surgeon establishes the infusion tubing, the operating room nurse and the anesthesiologist independently verify the information related to the islet cell expansion solution, including the patient’s name, gender, age, medical record number, bed number, blood type, diagnosis, specimen name, specimen dose, and expiration date, to ensure accuracy.

Once confirmed, a standard infusion set (without a filter) is used to start infusing the islet cell expansion solution as per medical instructions. The infusion begins at a slow rate of 15 drops per minute for 15 minutes. During this time, the operating room nurse records the patient’s vital signs every 5 minutes. If the patient’s vital signs remain stable, the infusion rate can be gradually increased to 60 drops per minute, and the operating room nurse continues to record the patient’s vital signs every 15 minutes until the infusion is completed.

Finally, the used islet cell expansion solution bag is stored for 1 week to facilitate subsequent record retrieval and tracing.

## 5. Outcome

**Figure 1.**
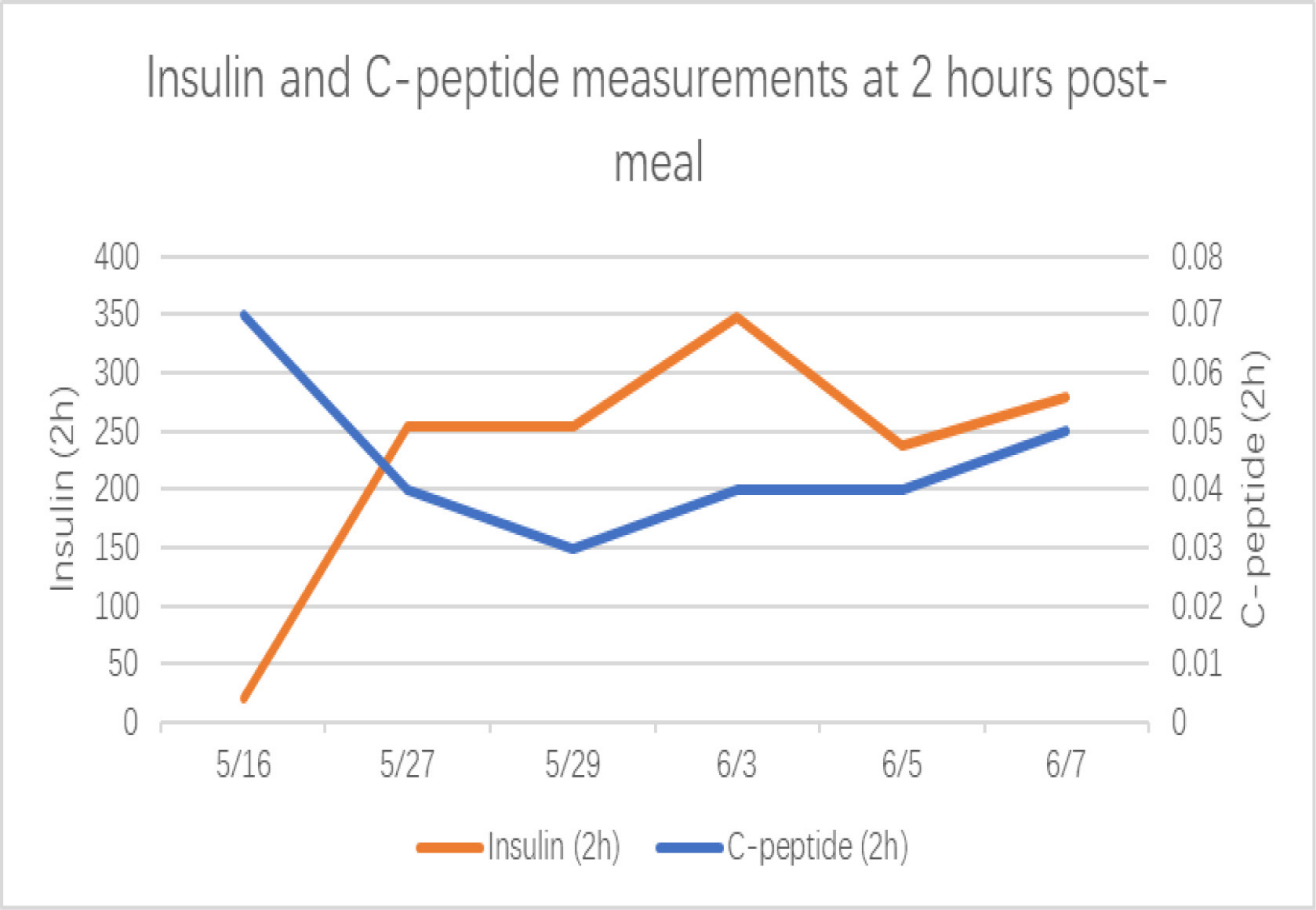
Insulin and C-peptide measurements 2 hours post-meal.

**Figure 2.**
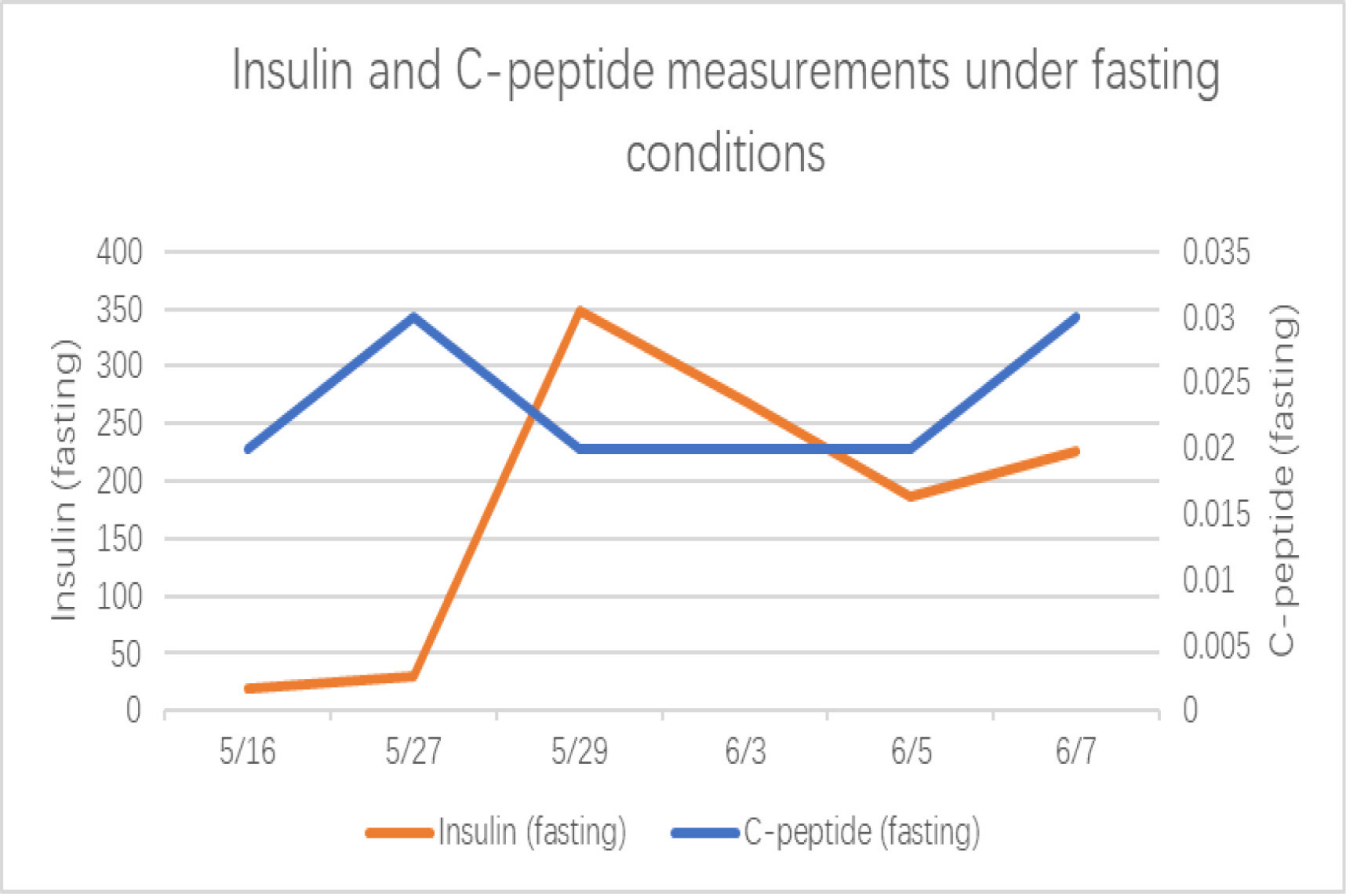
Insulin and C-peptide measurements under fasting conditions.

After meticulous treatment and nursing care, the patient was discharged from the hospital after 18 days of hospitalization. Figures 1 and 2 show insulin and C-peptide measurements at 2 hours post-meal and under fasting conditions, respectively. The patient has been successfully weaned off exogenous insulin therapy.

## 6. Conclusion

In this case, the patient experienced poor graft function after the initial islet transplantation due to various factors, necessitating a second procedure. Given the complexity of the patient’s condition and the subsequent challenges in surgical coordination, operating room nurses, as key members of the multidisciplinary team, needed to thoroughly understand the integrated treatment plan and develop corresponding nursing strategies. The successful completion of the surgery was achieved through the collaborative efforts of general surgeons, interventional radiologists, anesthesiologists, laboratory technicians, and operating room nurses, with each team member fulfilling their respective roles.

Prior to surgery, operating room nurses should provide personalized preoperative visits, conduct comprehensive risk assessments, and implement effective intervention measures based on the assessment results. Special attention should be given to enhancing infection control during the planned transplantation surgery and prioritizing the prevention and nursing of complications to ensure the patient’s safe return to the ward.

Through meticulous treatment and nursing care, the patient in this case was able to recover and be discharged from the hospital after 18 days, successfully weaned off exogenous insulin therapy. This outcome highlights the importance of multidisciplinary collaboration, comprehensive nursing plans, and effective preoperative and intraoperative measures in ensuring successful outcomes for patients undergoing complex surgeries such as islet transplantation.

## Acknowledgments

We express our sincere gratitude to all of the participants for their valuable collaboration.

## Author contributions

**Conceptualization:** Jianwen Yang, Xuefeng Qi.

**Data curation:** Jianwen Yang, Xuefeng Qi, Qin Xu.

**Formal analysis:** Xuefeng Qi.

**Funding acquisition:** Jianwen Yang.

**Investigation:** Jianwen Yang, Xuefeng Qi.

**Methodology:** Xuefeng Qi, Weiming Qian.

**Project administration:** Jianwen Yang, Xuefeng Qi.

**Writing** – **original draft:** Jianwen Yang, Xuefeng Qi.

**Writing** – **review & editing:** Jianwen Yang, Xuefeng Qi.
